# QT interval prolongation: clinical assessment, risk factors and quantitative pharmacological considerations

**DOI:** 10.1007/s10928-025-10010-x

**Published:** 2025-11-07

**Authors:** Verena Gotta, Birgit Donner

**Affiliations:** 1 Clinical Pharmacy, University Children’s Hospital Basel, Basel, Switzerland; 2 Pediatric Pharmacology and Pharmacometrics, University Children’s Hospital Basel, Basel, Switzerland; 3 Pediatric Cardiology, University Children’s Hospital Basel, Spitalstrasse 33, Basel, 4056 Switzerland

**Keywords:** QT interval, LQT syndrome, Ion channelopathy, Drug-induced QT prolongation, Pharmacovigilance

## Abstract

Prolongation of the QT interval in the ECG is a critical finding that signifies an extended duration from the onset of ventricular depolarization to the end of ventricular repolarization. It can predispose patients to life-threatening arrhythmias, such as Torsades de Pointes (TdP). Long QT syndromes (LQTS) are defined by mutations in ion channel genes, particularly those encoding cardiac potassium and sodium channels and are characterized by a significant risk for sudden cardiac death if untreated. However, besides these clearly defined entities various medications have been implicated in causing QT interval prolongation. There is increasing evidence for a genetically determined risk for drug-induced QT prolongation. In addition, due to numerous clinical factors influencing the QT interval, QT prolongation increases the risk of TdP particularly in multi-morbid patients necessitating vigilant monitoring in at-risk populations. This review gives an overview of mechanisms and conditions which induce QT prolongation, the clinical assessment of QT interval duration, thereby highlighting quantitative variations in measurement techniques and heart-rate correction, as well as in demographic interpretation of normal values. The risk of cardiac arrhythmia is discussed, in both patients with congenital LQTS and acquired QT prolongation, along with influencing pharmacokinetic/pharmacodynamic, non-pharmacologic and genetic risk factors for TdP. Finally, clinical implications for individual patient management, including risk-adapted drug-prescription and use of ECG monitoring to mitigate the risks associated with QT prolongation, are summarized. Understanding the interplay between pharmacokinetics, pharmacodynamics, genetic predisposition and co-morbidities is essential for optimizing treatment in the context of prolonged QT intervals, preventing adverse cardiovascular events, and improving cardiac safety. Comprehensive drug labelling regarding exposure-QT relationships and available pharmacovigilance data are important sources of information enhancing patient safety.

## Introduction

The QT interval on the electrocardiogram (ECG) represents the summation of action potentials in ventricular myocytes, comprising ventricular depolarization and repolarization [1, 2]. A prolonged QT interval is a surrogate of prolonged cardiac repolarization, which is a risk factor for potentially life-threatening Torsade de Pointes (TdP) tachycardias, and sudden cardiac death [3]. Prolongation of the QT interval can be congenital or acquired, is frequently drug-related, and is thus of concern both in the context of drug development and clinical drug use [4, 5]. Drug induced heart-rate corrected QT (QTc) interval duration of more than 500 ms predicts short term mortality independent of comorbidities [6].

A profound understanding of pharmacokinetic/pharmacodynamic (PK/PD) relationships and patient-specific pharmacodynamic risk factors can support development of safe dosing strategies for high-risk drugs. For example, the antiarrhythmic drug sotalol has been reported to induce in about 2.4% of patients Torsades de pointes (TdP) tachycardias in an exposure-dependent manner, with higher risks in women and patients with heart failure [7]. Model-informed drug development (MIDD) based on PK/PD considerations has allowed to formulate safe intravenous dosing strategies for sotalol and reduction of obligatory hospital stay for cardiac monitoring [8]. Also for non-antiarrhythmic drugs a thorough assessment of drug effects on the QTc interval is required and of importance for safe clinical drug use [9]. When studying clinical concentration-QTc relationships, a quantitative understanding of variability, including variations in measurement techniques, heart-rate correction, and other clinical influencing factors is of importance. Incorporation of preclinical findings, such as in vitro measured ion channel binding potency or in vivo concentration-QTc effect in preclinical species, can enhance mechanistic understanding of drug effects and variability [10, 11]. Furthermore, the relationship between quantified effects on the QTc interval and expected risk of life-threatening events need to be understood.

This review provides an overview on the electrophysiological basis of the QT interval, its clinical measurement and interpretation, considering various influencing factors and quantitative measures for risk assessment during clinical drug development and drug use.

### Electrophysiological basis of cardiac repolarization

**Fig. 1:**
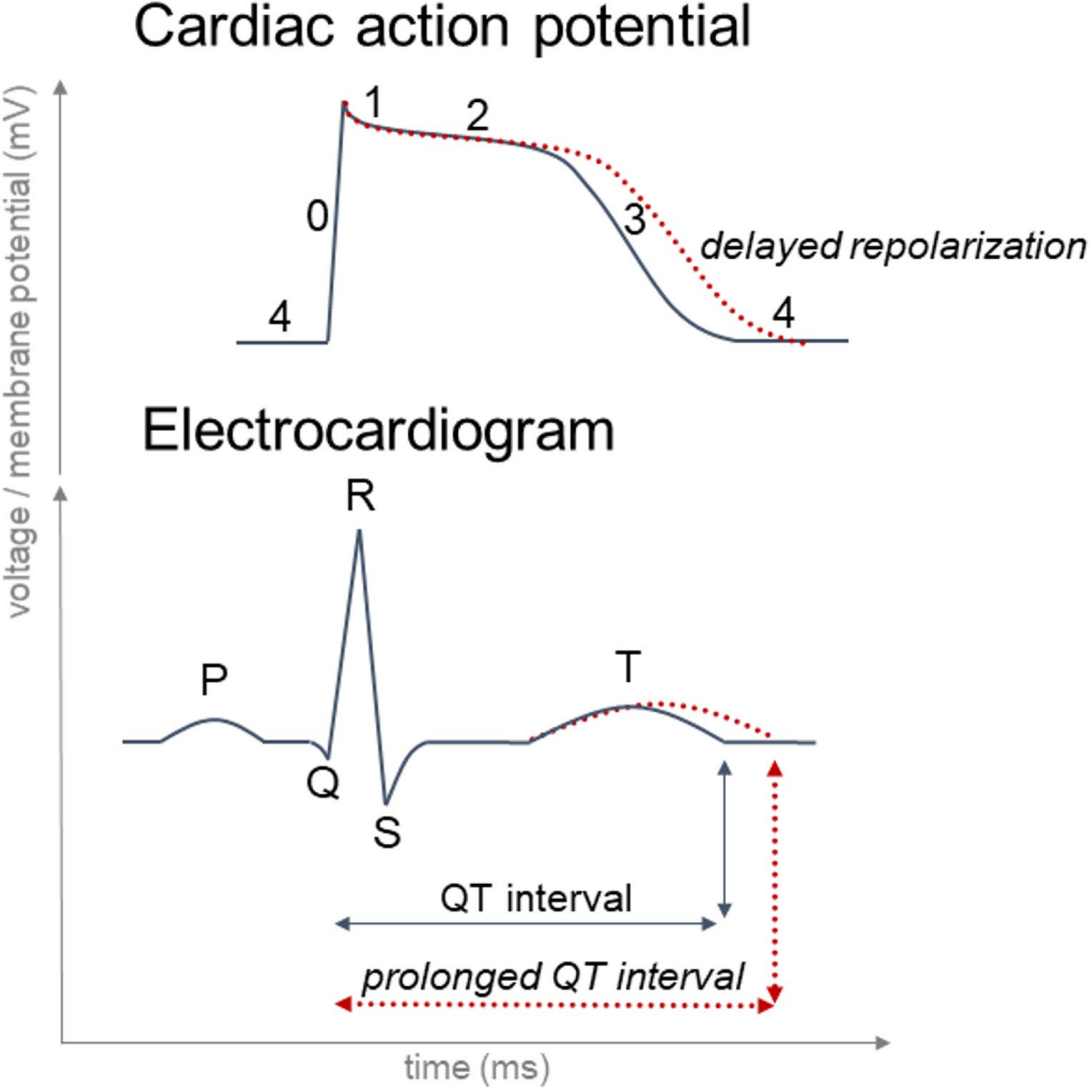
Schematic representation of the different phases of a cardiac action potential (ventricular myocytes) and correlation with segments on the electrocardiogram. Phase 0: depolarization. Phase 1–3: repolarization (details: see text). Phase 4: resting potential (diastole). Therefore, the QT interval represents the duration of ventricular depolarization and repolarization, a prolonged QT interval (dotted lines) might be associated with delayed repolarization, which is a risk factor for TdP and sudden cardiac death [3]

Cardiac repolarization is a tightly regulated process that can be subdivided into three phases (Fig. 1) with the involvement of different potassium and calcium currents finally restoring the negative resting membrane potential:

- Early Repolarization (Phase 1): Initiated by transient outward K⁺ currents (Ito), which cause a brief drop in membrane potential following depolarization.
- Plateau Phase (Phase 2): A balance between inward calcium (Ca^2+^) currents and outward K⁺ currents maintain a stable potential (reflected on the ECG as ST-segment).
- Late Repolarization (Phase 3): Dominated by delayed rectifier K⁺ currents (I_Kr_ and I_Ks_) and inward rectifier K⁺ currents (I_K1_), which restore the negative resting membrane potential (reflected on the ECG by the T-wave).

### Electrophysiological mechanisms that lead to Torsades de pointes (TdP) Tachycardias

TdP describes a polymorphic ventricular tachycardia with a distinctive fluctuating morphology of QRS complexes twisting around the baseline of the ECG. TdP arises due to a combination of prolonged repolarization, early afterdepolarizations, altered action-potential configuration and heterogeneous electrical recovery:

- The primary factor leading to TdP is prolongation of the ventricular action potential duration (APD), mainly due to delayed repolarization. Delayed repolarization is primarily determined by the rapid component of the outward repolarizing current (I_Kr_) in phase 3, mediated by the delayed rectifier potassium channel Kv11.1, frequently also called hERG channel (human ether-a-go-go related gene, hERG) [12]. Overall however, different outward (K^+^) currents and inward currents through sodium (Na^+^) and calcium (Ca^2+^) channels regulate the cardiac repolarization [13]. When these channels are blocked or dysfunctional, the repolarization process becomes prolonged, increasing the risk of early afterdepolarizations (EADs). EADs are abnormal depolarizations that occur during the repolarization phase of the action potential. They can trigger secondary electrical impulses. In addition, the presence of spatial and temporal dispersion of repolarization across the myocardium creates a heterogeneous environment conducive to reentrant circuits, leading finally to torsade de pointes [2].

Additional risk factors include electrolyte disturbances and certain medications (e.g. antimicrobial, antiarrhythmic, antipsychotic and antidepressant drugs), which impair the normal function of repolarizing currents, as discussed below.

### Methods to measure the QT interval duration

The QT interval duration can be measured automatically or manually. Manual measurement from 12-lead ECGs by expert cardiologists, recorded at a single time point (over seconds or minutes), ideally averaged from at least three cycles, still represents the gold-standard [9, 14]. Full- or semi-automated continuous monitoring using Holter-ECG, and increasingly also via smart digital devices are available [14, 15], which might have a role in long-term monitoring under drug therapy.

**Fig. 2:**
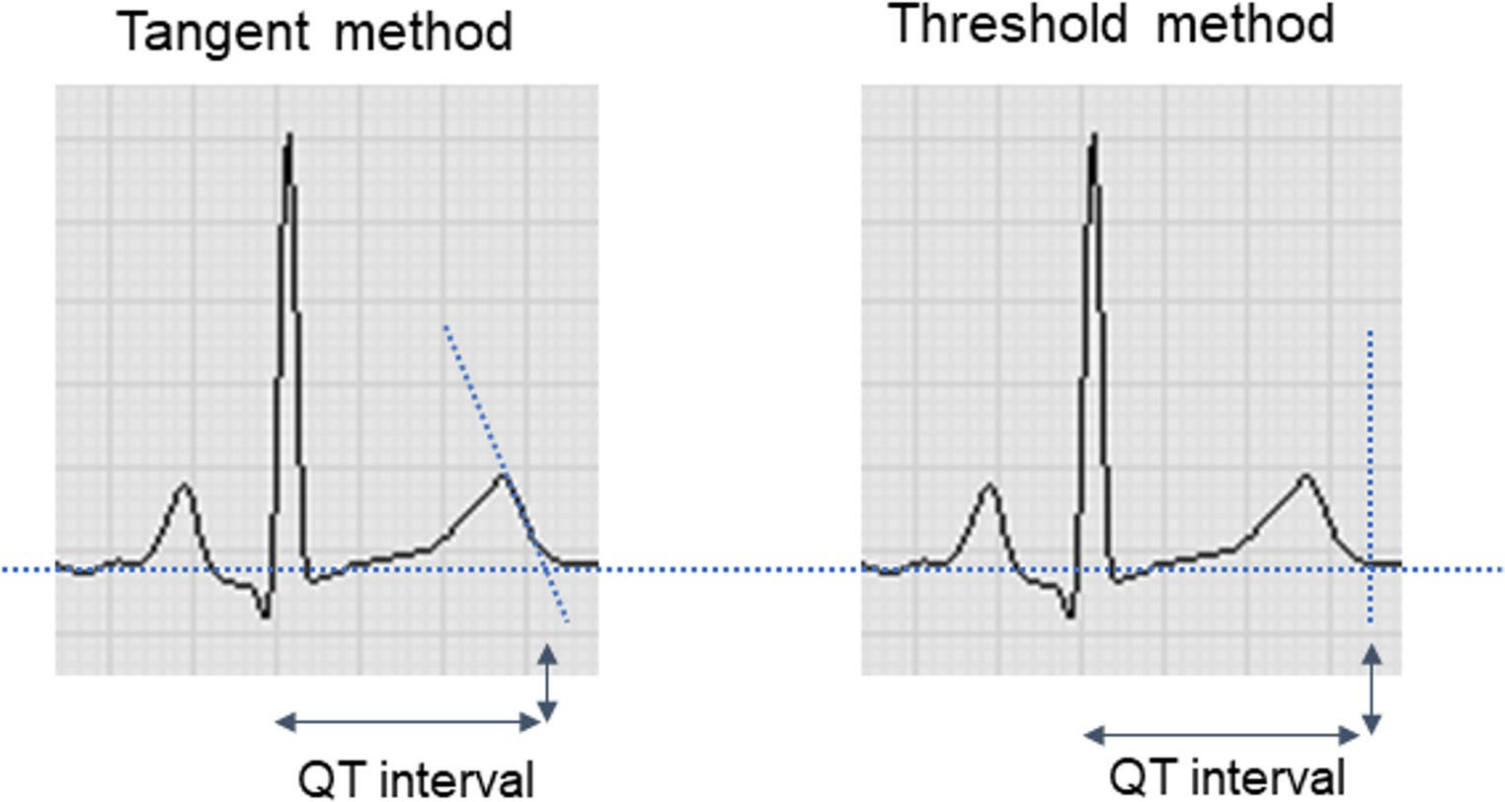
Schematic illustration of tangent and threshold method for measuring the QT interval duration

The tangent and threshold methods are two approaches for manually measuring the QT interval: the tangent method uses a line from the T wave peak through its steepest descending part to intersect the baseline, while the threshold method marks the T wave end where it reaches the baseline (Fig. 2).

Measurements using the tangent method reveal QT intervals that are approximately 10 ms shorter compared to using the threshold method, but are considered to have high intra- and inter-reader validity (95% limits of agreement ranging from ± 20 ms to ± 30 ms) in the context of diagnosis of congenital long-QT syndrome (LQTS) [16]. Therefore, the tangent method seems to be more consistent, reproducible, and less subjective, especially for ECGs with prominent U waves [17].

In the context of drug development, the choice of QT-interval measurement (fully manual, fully automated, semi-automated) may depend on the development stage. Whereas in clinical trials focusing on ECG safety assessment fully manual or semi-automated methods are recommended from a regulatory point of view, fully automated assessments may be adequate in late clinical trials involving drugs with negative QT/QTc study [18].

### The role of formulas for heart-rate correction of QT intervals

**Fig. 3:**
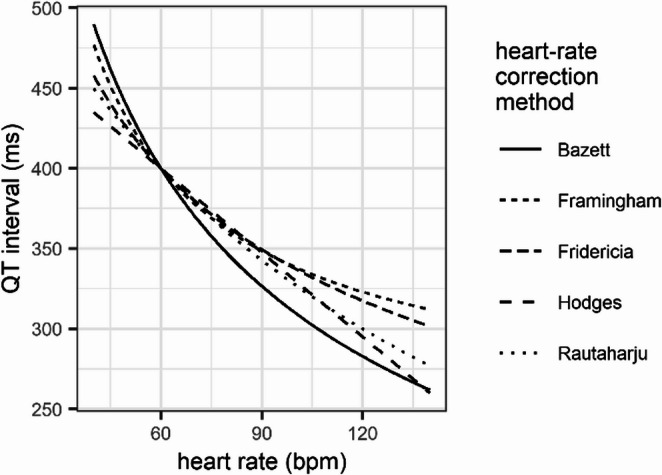
Predicted relationship between the QT interval duration and heart rate from different QTc prediction equations (Table 2)

The QT interval duration physiologically varies with heart rate, which is the reason for using correction equations to calculate the heart-rate corrected QT interval (QTc interval) [1]. The correction of QT interval for heart rate is crucial in assessing cardiac repolarization, but the choice of formula can significantly impact results. Out of about 25 different existing equations [19] commonly used formulae are depicted in Table 1, including early non-linear ones proposed by Bazett [20] and Fridericia [21] in 1920, as well as more recently linear equations proposed by Hodges [22], Framingham [23], Rautaharju [24] (Fig. 3) and nomograms [25]. The ideal equation would result in predicted QTc interval duration independent of heart rate (or RR interval, respectively), i.e. a horizontal scatter of QTc versus heart rate.

**Table 1:** Common equations used to calculate heart-rate corrected QTc intervals

Reference	Proposed equation
Bazett [20]	$\:QTc\:\left(ms\right)=QT/{(RR}^{1/2})$
Fridericia [21]	$\:QTc\:\left(ms\right)=QT/\left({RR}^{1/3}\right)$
Hodges [22]	$\:QTc\:\left(ms\right)=QT+0.00175\bullet\:(HR-60)\bullet\:1000$
Framingham [23]	$\:QTc\:\left(ms\right)=QT+1.54\bullet\:(1-RR)\bullet\:1000$
Rautaharju [24]	$\:QTc\:\left(ms\right)=QT\bullet\:(120+HR)/180$ (females) $\:QTc\:\left(ms\right)=QT\bullet\:(120+HR)/190$ (males)

*HR* heart rate (bmp), *RR* RR-interval (s) = 60/HR, *QT* QT-interval (ms), *QTc* heart-rate corrected QT-interval

Studies have shown that Bazett’s formula, while widely used, tends to overcorrect at slow heart rates and undercorrect at fast rates [26]. During exercise, Bazett and Hodges formulae overestimate QTc prolongation, while Fridericia and Framingham overestimate QTc shortening [27]. In contrast, the Rautaharju formula remains relatively constant across different heart rates, making it more suitable for evaluating QT intervals during rest and exercise [28]. However, for predicting TdP a cut-off of 477 ms has to be used [29]. Therefore, there is a trend to select the correction formula depending on specific clinical questions and further search for the optimal QTc calculation [19].

A standard visualization in concentration-QT modeling shows the QTc interval versus the RR interval to assess the (lack of) relation between the two measurements QTc and the RR interval. Ideally, QTc should be independent of the RR interval and therefore constant. Deviations indicate insufficient heart rate correction. It is noteworthy that such approach assumes that heart rate is not influenced by the drug itself [30]. Different methodologies have been proposed to handle QTc assessment the case where a drug has an impact on heart rate. Those include use of Holter bin analysis, dynamic QT beat-to-beat analysis, PK/PD modelling, and individual patient-specific correction formulas [31].

The ideal correction formula would indeed be patient-specific, based on individual QT-heart rate relationships [26]. This requires continuous (e.g. Holter ECG) or repeated ECG monitoring. and is clinically not applied as reference values have mainly been established based on population-based equations. In clinical studies, it may be feasible to use continuous or repeated ECG monitoring to establish individual correction equations - unless data are sparse, or when the heart-rate range to derive individual corrections (baseline measurements) does not cover the heart rate range observed under the studied drug [18].

### Normal QTc values depend on age and sex

Normal heart-rate corrected QTc interval values further vary with age and sex. The normal QTc interval is longest in the newborn, particularly during the first week after birth [32, 33]. During childhood, similar values are observed for boys and girls, then during adolescence a shortening is observed in adolescent boys [34], probably associated with an increase in testosterone [35]. As a result, in adults the normal QTc interval is approximately 12–15 ms shorter in young men compared to women, while after 40 years of age sex differences become smaller and then disappear in the elderly [1]. Table 2 displays routinely used age- and sex-dependent normal values of the QTc interval, that partly reflect these known demographic dependencies.

**Table 2:** Age- and gender dependent normal values of the QTc interval (adapted from [36])

Patient population	Normal value (ms)	Borderline value (ms)	Prolonged QTc interval (ms)
Newborns	< 450	450–470	>470
Children and adolescents	< 440	440–460	>460
Women	< 450	450–460	>460 [1]
Men	< 430	430–450	>450 [1]

### Circadian variation

Circadian variations in the QTc interval duration can be important and result from neurohumoral variations and diurnal changes in cardiomocyte-specific gene expression influencing the heart’s electrical properties. Briefly, highest QTc values are observed in the early morning around 3 AM [37, 38], and are ideally accounted for in clinical safety investigations assessing drug-induced QTc interval prolongation by time-matched placebo-controlled analysis [30]. Individual diurnal variations have been quantified by a mean daily amplitude of 24 ms (range 10 to 47 ms) [39]. Interestingly, diurnal patterns are also observed for cardiovascular diseases, including sudden cardiac death, which appears to peak in the early morning [40].

## Clinical relevance of QT interval prolongation

Drugs producing mean QT/QTc-interval prolongation of < 5 ms at supratherapeutic exposure have rarely been linked with significant cardiac risk [41]. In line with that, from a drug development perspective statistically significant average drug-induced QT/QTc-interval changes of >5 ms are set as threshold of regulatory concern [9]. Clinically, the diagnosis of congenital and acquired long QT syndrome (LQTS) mainly relies on absolute QTc values. Those values have been associated with relevant clinical outcomes in different patient populations as reviewed in the following. Furthermore, a description of clinical and pharmacotherapeutic management aspects will be provided, which depend on underlying pathophysiologic mechanisms.

### Congenital Long QT syndromes

#### Epidemiology and clinical significance

**Fig. 4:**
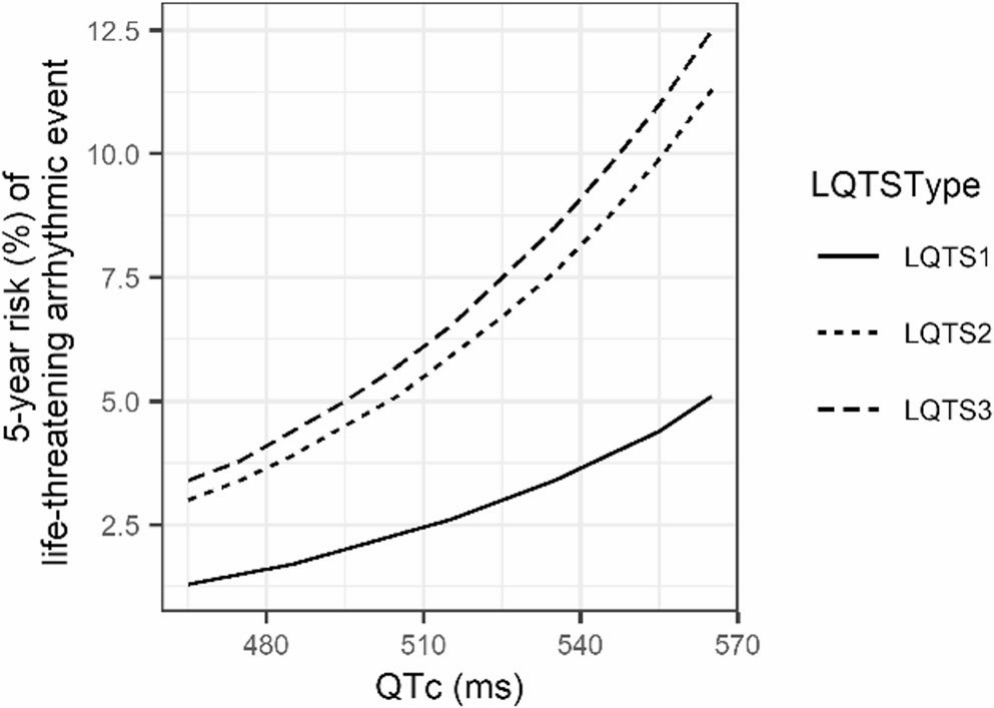
Relationship between QTc interval and 5-year risk of life-threatening arrhythmic events in congenital LQTS patients (sudden cardiac death, aborted cardiac arrest, and hemodynamically non-tolerated polymorphic ventricular tachycardia), according to data published by Mazzanti, et al. [45]

The prevalence of genetically determined congenital LQTS has been estimated at approximately 1:2000–2500 live births [42]. For untreated LQTS a cumulative incidence of cardiac arrest or sudden death events of 13% before the age of 40 years has been reported [43]. The probability of cardiac events significantly increases with longer QTc intervals, in symptomatic patients (e.g. those with tachycardias, or syncope), and varies with the genetic cause [42–44] (example shown in Fig. 4 [45]). Concealed LQTS has to be taken into account in any family history of sudden cardiac death.

#### Diagnosis and genetic testing

For diagnosis of LQTS ECG, clinical and/or genetic findings are used. According to current guidelines congenital LQTS is diagnosed clinically if QTc ≥ 480 ms in repeated 12-lead ECGs, or if there is a LQTS diagnostic score >3 (Table 3) [44].

**Table 3:** Modified long QT syndrome diagnostic score. A total score >3 is required for diagnosis of LQTS [44, 46]

Finding			Points
ECG	QTc	≥ 480 ms	3
		= 460–479 ms	2
		= 450–459 ms (in males)	1
		≥ 480 ms during 4th minute of recovery from exercise stress test	1
	Torsade de pointes		2
	T wave alternans		1
	Notched T wave in 3 leads		1
	Low heart rate for age		0.5
Clinical history	Syncope	With stress	2
		Without stress	1
Family history	Family member(s) with definite LQTS		1
	Unexplained SCD at age < 30 years in first-degree family		0.5
Genetic finding	Pathogenic mutation		3.5

*ECG* electrocardiogram, *LQTS* long QT syndrome, *SCD* sudden cardiac death

In addition, for improving the diagnosis of congenital LQTS in the context of above described variability of normal QTc values depending on age, sex, measurement technique, and applied heart-rate correction, a probability calculator has been developed (www.QTcalculator.org). It determines the probability that the calculated QTc interval belongs to a patient with genetically determined congenital LQTS or a non-LQTS patient, based on a large cohort of genotype-positive LQTS patients and genotype-negative family members as controls [16].

Furthermore, artificial intelligence (AI) driven approaches may enhance the detection of concealed LQTS by analyzing subtle patterns and features, such as T wave morphology, and help to identify patients with mostly normal QTc intervals but who are still at risk for sudden cardiac death [47–49].

Following clinical diagnosis of LQTS genetic counseling and testing is recommended to guide genotype-specific management (pharmacotherapy and avoidance of specific risk factors) and identify relatives at risk. Genetic testing has revolutionized the diagnosis and management of LQTS, with approximately 75% of affected individuals having an identifiable genetic cause. Although there are at least 17 genes associated with LQTS, mutations in the genes causal for LQTS subtypes 1–3 (LQTS1-3) account for 90% of positively genotyped cases (Table 4) [50]. These three most common genetic subtypes involve loss-of-function variants in the potassium channel genes *KCNQ1* (LQTS1) and *KCNH2* (hERG channel, LQTS2), and gain-of-function variants in the sodium channel gene *SCN5A* (LQTS3) [42]. Triggers for cardiac events vary between subtypes: whereas adrenergic activation plays a role for LQTS1 (particularly through exercise) and LQTS2 (particularly through emotional stress), cardiac events in LQTS3 more frequently occur at rest and during sleep [42, 44].

**Table 4:** Channel mutations in LQTS 1–3, physiological functions and gene-specific risk factors (adapted from [51] with modifications)

LQTS	Proportion of cases	Gene	Channel/Current	Physiological function during repolarization	Current change due to mutation	Risk factor for TdP
LQTS1	~ 50%	*KCNQ1*	K_V_7.1/I_Ks_	Phase 3 repolarization	Reduced I_Ks_ Loss of function	Jump in cold water, strenuous exercise
LQTS2	~ 40%	*KCNH2* (human ether-a-go-go-related gene, hERG)	K_V_11.1/I_Kr_	Phase 3 repolarization	Reduced I_Kr_ Loss of function	Loud unexpected noise: Alarm clock/telephone ringing, emotional stress
LQTS3	~ 10%	*SCN5A*	Na_V_1.5/I_Na_	Phase 0 depolarization of action potential	Increased I_Na_ Gain of function	Bradycardia at sleep/rest

I_Ks_ slow component of the delayed outward rectifier potassium channel

I_kr_ rapid component of the delayed outward rectifier potassium channel

I_Na_ sodium voltage-gated channel alpha subunit 5

#### Management

Non-selective beta-blockers (nadolol or propranolol) significantly reduce the risk of arrhythmic events in LQTS1 and LQTS2, whereas sodium channel blockers (mexiletine) are indicated in LQTS3 and LQTS2 patients at high risk [52, 53]. Implantable cardioverter-defibrillator (ICD) are indicated in patients with tachycardia despite of medication. For prevention, gene-specific risk factors (Table 4) and QT prolongating medications should be avoided (www.crediblemeds.org). Electrolyte disturbances (i.e. hypocalcemia, hypomagnesemia and hypokalemia) should be rapidly diagnosed and corrected. Research efforts are focused on discovering new therapeutic targets and strategies to modify both congenital and acquired QT prolongation. One promising avenue is the development of hERG activators for the treatment of LQTS2 [54].

## Acquired QT prolongation: drug and patient specific risk factors

### Acquired LQTS is frequently drug-associated, and may be augmented with patient-specific factors

#### Drug-induced proarrhythmia

A well studied mechanism of drug-induced QTc prolongation involves hERG channel inhibition, by binding to the pore of the channel or interfering with channel trafficking. Inhibition of the rapid component of the delayed rectifier potassium current (IKr) prolongs cardiac repolarization and increases the risk for EADs due to activation of inward depolarizing currents (L-type calcium channels or sodium- calcium exchange current), which can ultimately progress to TdP [2]. This is the reason why new drugs are now – since introduction of ICH S7B guideline for nonclinical evaluation of the potential for delayed ventricular repolarization by human pharmaceuticals in 2005 – tested early on for hERG inhibition. In addition, drugs reducing the currents of I_Ks_, late sodium or L-type calcium currents can induce QTc prolongation [13]. These effects are further augmented if different cardiac ion channels are involved. However, QT prolongation and antiarrhythmic properties might be combined in one drug. Characterizing the interaction with other repolarizing channels than hERG need to be considered in the risk assessment for TdP, as blocking calcium and/or sodium inward currents may reduce the risk for EADs [55]. For example, the cardiac drug ranolazine, a sodium channel blocker, has been shown to be antiarrhythmic despite hERG channel inhibition and observed concentration-dependent QTc prolongation [56].

#### Epidemiology and clinical significance

Acquired LQTS has a high prevalence of >20% in hospitalized patients [57–59]. Although TdP tachycardia in this setting are relatively rare, they may be fatal [57, 60]. In a tertiary hospital population TdP incidence was estimated as 0.16‰/year, which would correspond to approximately 173 possibly lethal TdP-cases per year nationally, in a small country like Belgium, in this high risk population with diverse health problems and diseases. Approximately half of the events were attributed to acquired LQTS and mostly involved treatment with at least one QT-prolonging drug [60]. Prolonged repolarization represents an additional dose-dependent risk factor, especially in elderly patients or patients with an underlying disease (Table 5).

**Table 5:** Risk factors for torsade de pointes (TdP) (adapted from drew et al. [61] with modifications)

Clinically recognizable risk factors
Patient demographics
• Advanced age
• Female sex
QTc >500 ms
LQT2-type repolarization: notching, long Tpeak–Tend
Use of QT-prolonging drugs
• Concurrent use of more than one QT-prolonging drug
• Rapid infusion by intravenous route
• Impaired hepatic drug metabolism (hepatic dysfunction or drug-drug interactions)
• Impaired kidney function
Electrolyte imbalances
• Hypokalemia
• Hypomagnesemia
• Hypocalcaemia
• Treatment with diuretics
Systemic disease
• Congestive heart failure
• Cardiomyopathy
• Myocardial infarction
• Thyroid dysfunction, mainly hypothyroidism
• Cardiac autonomic neuropathy
• (nocturnal/undiagnosed) hypoglycemia in diabetes mellitus
• Inflammation and impaired immunity
• Obesity
Arrhythmia
• Sinus bradycardia, (incomplete) heart block, pauses
• Premature ventricular complexes leading to short-long-short cycles
**Clinically silent risk factors**
(concealed) congenital LQTS
Genetic polymorphisms (“reduced repolarization reserve”)

*QTc* heart-rate corrected QT interval, *LQTS* long QT syndrome, *LQT2* congenital long QT syndrome type 2

**Fig. 5:**
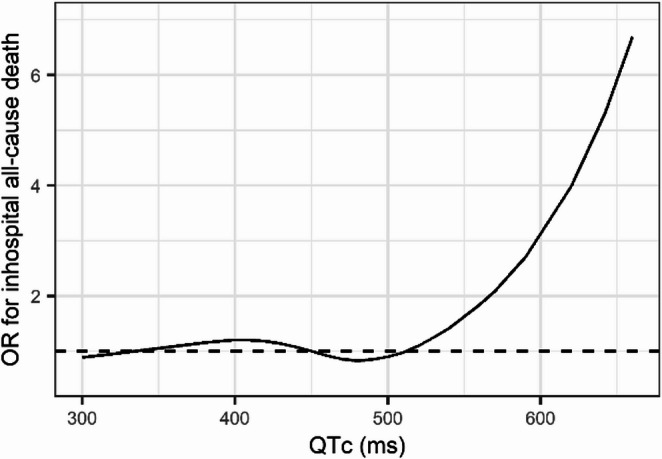
In-hospital all-cause mortality in patients with acute heart failure as a function of magnitude of QTc interval at emergency department admission, according to data published by Miro, et al. [63]. OR: odds ratio. Dotted horizontal line: OR = 1 (no difference)

For example, in elderly patients the risk for sudden cardiac death rises already approximately 2–3 fold in the range of borderline and abnormally prolonged QTc, respectively, after adjusting for demographic and disease-related risk factors [62]. Also in patients admitted for acute heart failure, there is a clear relationship between QTc interval and in-hospital all-cause mortality (Fig. 5) [63].

#### Diagnosis and risk factors

Normal QTc reference values (Table 2) are used in diagnosing acquired QT prolongation. Identification of its cause and reducing risk factors for TdP is of high clinical importance.

An analysis of TdP cases associated with noncardiac drugs [64] has shown that most patients have one or more other known risk factor for TdP [61] (Table 5), including female sex, heart disease (myocardial infarction, heart failure, valvopathy or cardiomyopathy), hypokalemia, supra-therapeutic dose/exposure (including missing adjustment for kidney or liver failure, or drug interactions), and familial history of long QT syndrome [64].

There are numerous risk factos for TdP, many of which are explained by their contribution to QTc-interval prolongation [65] (Table 5). For example thyroid dysfunction not only in hypothyroidism but also in hyperthyroidism seems to deregulate the expression of genes involved in cardiac repolarization thereby prolonging the QT interval and increasing electrical instability [66, 67]. The risk associated with diabetes mellitus is multifactorial and appears to be triggered by hypoglycemia-associated hERG inhibition, adrenergic activation, hypokalemia and associated QTc prolongation, as well as by disease-related factors such as cardiac autonomic neuropathy [38, 68]. Inflammation and immune reactions induce or enhance electrical instability in acquired and congenital LQTS, presumably mediated by direct effects of cytokines and autoantibodies on cardiomyocyte ion channel expression and function [65].

#### Management

Management of acquired LQTS includes optimizing drug treatment and identification and correction of reversible causes, mainly specifically treating the underlying disease thereby minimizing risk factors. Any precipitating drug should be discontinued and/or substituted by an alternative, and metabolic abnormalities (hypokalemia, hypomagnesemia) should be corrected timely., Especially the serum potassium level should be kept in the high normal range (shortens QT interval, reduces QT dispersion) [2]. The administration of prophylactic magnesium is still controversial. The assessment of individual risk will determine the need for continuous ECG monitoring, which should be applied until normalization of the QT interval.

The treatment of persistent TdP tachycardia includes electrical cardioversion and intravenous administration of magnesium. The mechanism by which intravenous magnesium (agent of choice for acute treatment of TdP even in the absence of hypomagnesia [44]) prevents recurrences of TdP is not fully elucidated, but may be explained by blockage of sodium or calcium currents [2]. Antiarrhythmic drugs such as quinidine, lidocaine or amiodarone can be used, with caution, since they also can worsen the arrhythmia. In case of bradycardia isoproterenol or pacemaker stimulation are effective.

### Importance of understanding and controlling pharmacokinetic variability

Drug-induced QTc prolongation is considered a concentration-dependent effect, as supported by preclinical investigations of receptor occupancy (e.g. hERG ion channel binding). Hence understanding and controlling pharmacokinetics and associated variability – through application of MIDD during drug development or careful choice of mode of administration (e.g. duration of infusion, extended versus immediate release oral formulation) and clinical dose adjustment (e.g. for age, comedication, renal function) – can help to mitigate the risk of clinically significant QTc prolongation. For example, PD modeling of ondansetron has found a direct linear concentration-QTc relationship with doses up to 32 mg. Integration with PK has helped to redefine age-dependent intravenous dose limits for cancer patients, based on model-predicted QTc at maximal plasma concentration (C_max_) [69]: a maximum dose of 16 mg in adults < 75 years is recommended, whereas dose should be limited to 8 mg in elderly patients ≥ 75 years due to reduced clearance and increased exposure, both should be infused over a minimum duration of 15 min to limit C_max_ and associated mean QTc prolongation to ≤ 10 ms. The risk of QTc prolongation is significantly lower under equivalent oral doses due to lower C_max_. Lower intravenous doses of 4 mg, given to prevent post-operative nausea and vomiting, may be given as injection. Another MIDD example is sotalol [8] (see introduction). General examples underlining importance of drug exposure are the market withdrawal of terfenadine and cisapride due to TdP cases occurring under therapeutic doses, which was attributed to drug-drug interactions resulting in elevated plasma exposure [41] and significant QTc prolongation [70].

### Importance of pharmacodynamic understanding

Whereas clinical PK/PD modelling can help to characterize and quantify drug exposure and corresponding QTc effects under different conditions, preclinical in vitro experiments are required for the mechanistic understanding of drug-induced QTc prolongation and its proarrhythmic risk. Such in vitro experiments include for example the patch clamp assay for assessment of in vitro hERG/I_Kr_ block (with associated half-maximum inhibitory concentration, IC50) and interaction with other ion channels, or the induced pluripotent stem cells (iPS)-derived cardiomyocyte assay [71]. The ratio of hERG IC50 to unbound Cmax has been shown to correlate with TdP, and a cut-off value of < 30 has been proposed to be associated with increased risk [72]. However, interaction with other ion channels needs to be considered in the risk assessment as discussed above (example ranolazine). Comprehensive in vitro data can be aggregated by in silico pharmacodynamic modelling of varying complexity and combined with in vivo PK data to support drug development decisions, reduce requirement of animal experiments, and better understand the proarrhythmic risk [73].

### Inter-individual pharmacodynamic variability in drug-induced QTc prolongation

Quantifying inter-individual variability in sensitivity to drug-induced QTc prolongation is of great clinical interest. The pharmacodynamic variability in the drug concentration -QTc interval relationship is multifactorial, including demographic and ethnic/genetic differences [74, 75], respectively, and remains only partially understood. For example, the drug concentration-QTc relationship in neonates and women is steeper compared to infants and men, respectively, potentially due to age- and sex related differences in density of ion channel expression [11], and may even show circadian variations [76]. Therefore, also a substantial proportion of the pharmacodynamic variability in the relationship between QTc interval duration and TdP risk remains unexplained.

### Genetically determined risk for drug-induced QT prolongation

In recent years, growing attention has been directed toward understanding the pharmacogenetics of drug-induced LQTS (diLQTS). Genetically determined risk for diLQTS is influenced by variations in genes that regulate cardiac ion channels (resulting in pharmacodynamic variability) and drug metabolism (resulting in pharmacokinetic variability) [75]. Certain genetic variants can reduce the heart’s “repolarization reserve,” making individuals more susceptible to QTc prolongation when exposed to specific medications. Examples for pharmacodynamic variability are genetic variants in genes such as *KCNH2*, *KCNE1*, and *KCNE2* that directly affect potassium channels, impairing the rapid delayed rectifier current I_Kr_ critical for cardiac repolarization [77]. Mutations in genes like *NOS1AP* (encoding a nitric oxide synthase adaptor protein) [75] influence ion channel function, thereby modifying the risk for drug-induced QT prolongation [75]. Genetic susceptibility due to pharmacokinetic alterations are mainly known from polymorphisms in the ATP-binding cassette B1 gene (ABCB1) encoding the P-glycoprotein and genetic variations in cytochrome P450 substrates. In addition, there is an increasing number of genes associated with predisposition for drug induced QT prolongation by genome-wide association studies [75]. Individuals with normal baseline QT intervals but this genetic predisposition show extreme QT prolongation under certain drugs or even TdP. Genetic screening could help identify at-risk patients and personalize treatment, improving drug safety and reducing adverse cardiac events [77]. 

## Considerations in drug prescription

In general, QTc-prolonging drugs are contraindicated in patients with congenital LQTS or baseline QTc >500 ms and have to be used with caution when preexisting acquired QT prolongation is known [44]. Also the drug-specific risk potential has to be considered [55].

Special caution is required in the prescription of anti-arrhythmic drugs associated with QT prolongation to patients with acquired prolonged QTc intervals, due to their inhibition of specific ion channels responsible for heart repolarization (class 1 A, e.g. quinidine: inhibition of fast sodium channel, class III, e.g. amiodaroneor sotalol: inhibition of potassium/hERG-channel, class IV, e.g. verapamil or diltiazem: inhibition of calcium channels) [2, 78], resulting in a significant risk of TdP ventricular tachycardia. In patients with specific risk factors for TdP (Table 5) treatable causes should be compensated before starting the medication or if not feasible, their use should be limited to strong indications under close ECG-monitoring. Monitoring during treatment requires preferentially ECG at baseline, after 1 day, and after 1–2 weeks of initiation (or broadly speaking at pharmacokinetic steady-state), or after increasing drug dose.

As drug-induced QT-prolongation is considered mainly a concentration-dependent effect, monitoring can be supported by drug concentration measurements (where feasible e.g. in the context of suspected drug-drug interactions), and should involve surveillance of renal/hepatic function in order to timely adjust dose – or to change treatment to a different drug with lower TdP potential [44]. Many noncardiac drugs (certain psychiatric medications such as haloperidol or chlorpromazine, antiinfectives such as macrolides or quinolones) go along with a significant risk of QTc prolongation and TdP [2, 64]. In contrast to these small molecules (< 800–1000 Da), which mainly bind to the hERG channel intracellularly [11], biologics like monoclonal antibodies do generally not cause direct QTc interval prolongation [79]. This may be mainly explained by their specific target binding, and possibly also by their large molecular size limiting distribution to the extracellular space [80]. Indirect effects through their pharmacological action may however be possible, as the example of denosumab-induced hypocalcemia in the context of osteoporosis treatment shows [79].

In the prescription process for at-risk patients comparative risk assessment of drug-related TdP potential can be a challenging task. To support clinical decisions different databases and tools [81] are available. In practice, the database “QTDrugs” list (available on www.CredibleMeds.org) is frequently consulted. This list comprises over 220 drugs, which are categorized based on standardized review and causality assessment regarding association with QT prolongation and TdP (risk categories: known risk of TdP, possible risk of TdP, conditional risk, no risk) [82]. A good correlation between these risk categories and QTc >500 ms or a change of >60 ms from baseline 24 h after hospital admission has recently been confirmed, where highest risk has been found for drugs with “known risks” [83]. The risk is further increased in the context of pharmacokinetic drug-drug interactions, as well as in the context of use of multiple QT-prolonging drugs (i.e. additive pharmacodynamic interactions) [83]. It is remarkable that a significant proportion of drug labels of commonly used drugs categorized as “known TdP risk” however lack appropriate information [84]. 

For newer drugs, published results of the thorough QT study with concentration-effect analysis [9] can enhance clinical risk assessment and management. Understanding the temporal pharmacokinetic/pharmacodynamic relationship of different drug combinations can be used to optimize timing of drug administration (especially in the context of multiple drug use), the mode of drug administration (e.g. switch from fast i.v. injection to longer infusion times or oral treatment, to avoid high peak concentrations), and to evaluate the importance of dosing in the given clinical context. For instance, dosing of psychotropic medications varies widely depending on indication, and as QTc prolongation is concentration- and hence dose-dependent [85] this consideration can be taken into account in the risk-benefit assessment. Targeted pharmacovigilance analyses comparing the risk of different drugs for a given indication quantitatively (e.g. serotonine reuptake inhibitors for depression [86]) can further promote an individualized drug selection.

Finally, also individual patient-specific factors must be considered in each risk assessment [81], which can be supported by calculating a validated QT risk score predicting QTc >500 ms or a change of >60 ms from baseline [82, 87, 88]. 

## Conclusions

The risk of QT prolongation and subsequent TdP is multifactorial, involving disease-related, individual, genetic, and pharmacological factors. Understanding these risk factors quantitatively, while taking into account measurement techniques of QTc intervals, is crucial for identifying high-risk patients and guide appropriate drug management, including drug selection and handling. This will allow implementing preventive strategies to decrease the risk of potentially fatal cardiac events.

## Data Availability

No datasets were generated or analysed during the current study.
